# Efficient generation of endogenous protein reporters for mouse development

**DOI:** 10.1242/dev.197418

**Published:** 2021-06-29

**Authors:** Daniel O'Hagan, Robin E. Kruger, Bin Gu, Amy Ralston

**Affiliations:** 1 Department of Biochemistry and Molecular Biology, Michigan State University, East Lansing, MI 48824, USA; 2 Reproductive and Developmental Sciences Training Program, Michigan State University, East Lansing, MI 48824, USA; 3 Department of Obstetrics, Gynecology and Reproductive Biology, Michigan State University, East Lansing, MI 48824, USA; 4 Institute for Quantitative Health Science and Engineering, Michigan State University, East Lansing, MI 48824, USA

**Keywords:** Blastocyst, Immunofluorescence, Knock-in, Mouse, Reporters, Split fluorescent protein

## Abstract

Fluorescent proteins and epitope tags can reveal protein localization in cells and animals, yet the large size of many tags hinders efficient genome targeting. Accordingly, many studies have relied on characterizing overexpressed proteins, which might not recapitulate endogenous protein activities. Here, we present two strategies for higher throughput production of endogenous protein reporters in mice, focusing on the blastocyst model of development. Our first strategy makes use of a split fluorescent protein, mNeonGreen2 (mNG2). Knock-in of a small portion of the *mNG2* gene, in frame with gene coding regions of interest, was highly efficient in embryos, potentially obviating the need to establish mouse lines. When complemented by the larger portion of the *mNG2* gene, fluorescence was reconstituted and endogenous protein localization faithfully reported in living embryos. Our second strategy achieves in-frame knock-in of a relatively small protein tag, which provides high efficiency and higher sensitivity protein reporting. Together, these two approaches provide complementary advantages and enable broad downstream applications.

## Abstract (precis)

**Summary:** We developed two complementary approaches in preimplantation mouse embryos, to enhance the efficiency of detecting endogenous proteins *in vivo*.

## INTRODUCTION

Mouse models are essential tools for research to uncover human disease mechanisms. To produce new mouse lines, embryos are collected and genetically manipulated during the preimplantation stage, and are then transferred to surrogate mothers for gestation. Thus, preimplantation embryos are the starting point for many studies. Within preimplantation, the blastocyst stage of development is also an alluring model in its own right. This is in part because the blastocyst provides technical advantages, including optical transparency, the capacity to develop *ex vivo* in a cell culture incubator, and the ease of collecting dozens of embryos at a time. These properties have enabled discovery of the molecular mechanisms of the first steps in mammalian development. Moreover, embryonic stem cells (ESCs) are derived from blastocysts, providing additional models for basic and applied research. Thus, technological advances using the blastocyst can impact broader areas of biomedical research.

One powerful approach to elucidating the molecular mechanisms of development and disease has been live imaging of fluorescent reporters *in vivo*, which enables time-resolved analysis of gene expression at the cellular level (Nowotschin and Hadjantonakis, 2014). Live imaging of gene expression *in vivo* is often achieved by knocking in genes encoding green fluorescent protein (GFP) and other fluorescent proteins downstream of gene promoters, to create gene reporters. This approach requires establishing and breeding new mouse lines. An alternative method of protein detection is to use antibodies to localize endogenous proteins, for example by immunofluorescence. However, immunofluorescence does not allow visualization of dynamic processes. Moreover, identification of reliable and specific antibodies can also be time intensive and, for some antigens, may not exist.

Our goal was to help overcome some of these challenges by developing an alternative, streamlined pipeline for the detection or screening of endogenous proteins *in vivo*. We focus on preimplantation mouse embryos, and present two complementary approaches to enhance the efficiency of detecting endogenous proteins *in vivo*. We provide guidelines for implementation of these approaches in broader experimental settings.

## RESULTS

### A mouse line to enable *in vivo* implementation of a split fluorescent protein

**Fig. 1.**
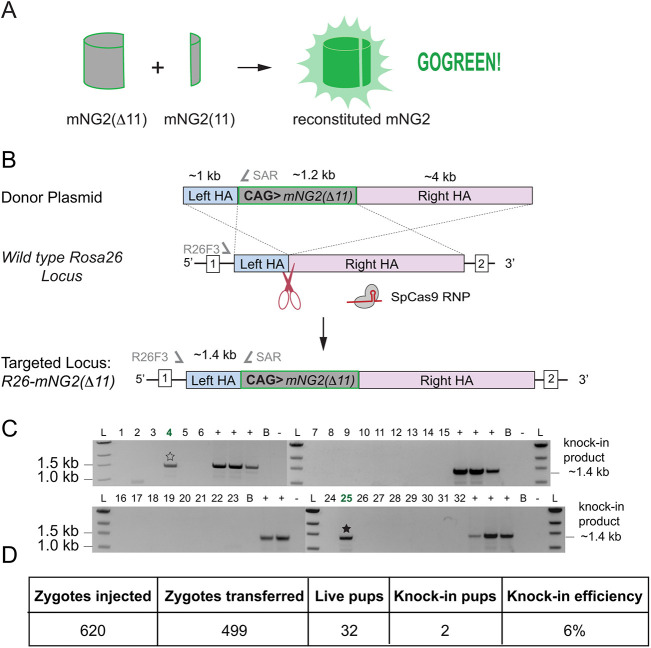
**A mouse line for fluorescence complementation *in vivo.*** (A) Deletion of mNG2(11), the 11th beta-strand of the fluorescent protein mNeonGreen2 (mNG2), eliminates its fluorescent properties. However, complementation by co-expression of *mNG2(Δ11)* and the 11th beta-strand *mNG2(11)* enables non-covalent association of the two proteins and reconstitution of the mNG2 fluorescent properties. (B) Strategy for CRISPR/Cas9 knock-in of the *mNG2(Δ11)* expression construct into the mouse *Rosa26* (*R26*) locus. Sequence of single-guide RNA (sgRNA), location of genotyping primers (*R26F3* and *SAR*) and predicted Cas9 cut site are shown. HA, homology arm; RNP, ribonucleoprotein. (C) PCR genotyping of tail tip biopsies from offspring born following zygote injection of CRISPR/Cas9 reagents to target the *CAG-mNG2(Δ11)* expression construct to the *R26* locus in zygotes. Successful homologous recombination suggested by PCR amplification of a 1.389 kb band from genomic DNA. B, C57BL/6 wild-type genomic DNA; L, DNA ladder; +, positive control (targeted embryonic stem cells); −, negative control (no DNA template). Numbers indicate individual mice screened by PCR; stars indicate potential founders. (D) Summary of *R26* targeting with *mNG2(Δ11)* expression construct.

Like GFP, the yellow-green, monomeric fluorescent protein mNeonGreen (mNG), derived originally from the marine invertebrate *Branchiostoma lanceolatum*, is an 11-stranded beta-barrel, but is up to three times brighter than GFP (Shaner et al., 2013). The mNG derivative, mNG2, can be split into two separate coding units, mNG2(Δ11), which lacks the 11th beta-strand, and mNG2(11), which is the 11th beta-strand (Feng et al., 2017). Individually, the two resulting proteins lack appreciable fluorescence. However, when the larger protein mNG2(Δ11) is complemented by the 16-amino acid mNG2(11), fluorescence is reconstituted (Fig. 1A), and the two proteins are capable of self-assembly through non-covalent intermolecular interactions (Cabantous et al., 2005).

We sought to make use of this fluorescence complementation strategy to evaluate localization of endogenous proteins in mouse embryos because we reasoned that tagging endogenous proteins with the smaller, 16-amino acid *mNG2(11)* coding region would be more efficient than knocking in the full-length gene encoding the full-length, 236-amino acid fluorescent protein. Then, to provide the complementary protein, we aimed to establish a mouse line capable of constitutive expression of *mNG2(Δ11)*. Our goal was to introduce an expression construct including cytomegalovirus enhancer, chicken beta-actin promoter, rabbit beta-globin splice acceptor (CAG) sequences and the *mNG2(Δ11)* coding region into the *Rosa26* (*R26*) locus by homologous recombination (Fig. 1B), which would enable constitutive, ubiquitous expression of *mNG2(Δ11)* throughout mouse tissues and development (Friedrich and Soriano, 1991). However, prior to attempting knock-in in mouse zygotes, we first established an *R26-mNG2(Δ11)* ESC line using a CRISPR/Cas9-mediated knock-in strategy (Chu et al., 2016) (see Materials and Methods). These *R26-mNG2(Δ11)* ESCs provided a renewable source of positive control genomic DNA for subsequent experiments.

To produce a mouse line capable of expressing *mNG2(Δ11)*, we subsequently introduced the *mNG2(Δ11)* expression construct into the *R26* locus in zygotes, following the strategy we had used in ESCs. Injected zygotes were transferred to recipient females, allowed to gestate, and then founder mice carrying *mNG2(Δ11)* were identified by PCR genotyping (Fig. 1C,D) and genomic sequencing. A single founder mouse was then expanded and bred to homozygosity to establish *R26-mNG2(Δ11)*/*R26-mNG2(Δ11)* mice. In principle, providing *mNG2(11)* in trans to *R26-mNG2(Δ11)* would lead to reconstitution of the fluorescent protein. For simplicity, we called this the GOGREEN system.

**Fig. 2.**
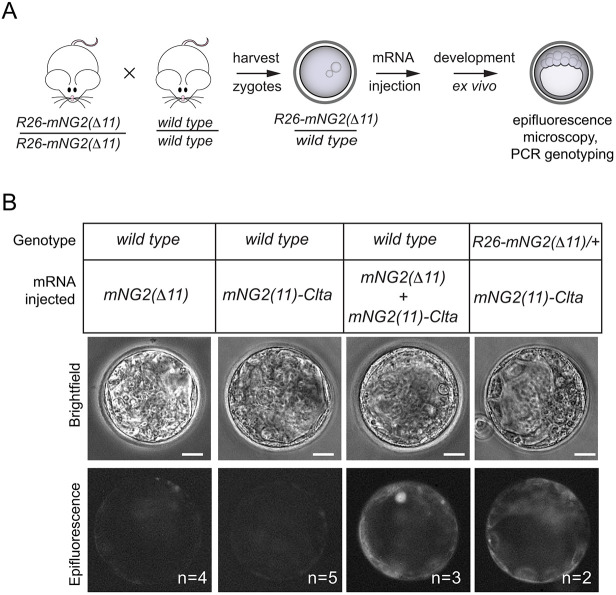
**Fluorescence complementation *in vivo* using the GOGREEN system.** (A) Strategy for testing fluorescent complementation in early embryos. Zygotes carrying *mNG2(Δ11)* were harvested and then injected with mRNA encoding mNG2(11)-tagged clathrin (CLTA). Zygotes were subsequently cultured *ex vivo* to later stages and fluorescence examined in individual embryos. Individual embryo genotypes were determined by PCR. In control experiments, zygotes were produced harvested from wild-type parents. (B) Fluorescence reconstitution can be detected by epifluorescence. Negative controls, wild-type embryos injected with mRNA encoding only *mNG2(Δ11)* or *mNG2(11)*-tagged *Clta* exhibit background levels of fluorescence (columns 1 and 2); positive control, wild-type embryos co-injected with *mNG2(Δ11)* and *mNG2(11)*-*Clta* exhibit reconstituted fluorescence (column 3); test of *R26-mNG2(Δ11)* mice, heterozygous knock-in embryos injected with mRNA encoding *R26-mNG2(11)-Clta* also exhibit reconstituted fluorescence above background (column 4). *n*, number of embryos evaluated in the experiment. Scale bars: 20 µm.

As an initial evaluation of the GOGREEN system, our first test was to determine whether we could detect fluorescence complementation in embryos by epifluorescence microscopy (Fig. 2A). For this test, we generated mRNA encoding *mNG2(11)-*tagged clathrin, light polypeptide (*Clta*). For negative controls, mRNA encoding either *mNG2(Δ11)* or *mNG2(11)-Clta* were injected individually into wild-type embryos (Fig. 2B). For a positive control, wild-type zygotes were co-injected with mRNAs encoding both *R26-mNG2(Δ11)* and *mNG2(11)-Clta*, and these exhibited greatly elevated fluorescence over both negative controls. Finally, *R26-mNG2(Δ11)*/+ zygotes were injected with mRNA encoding *mNG2(11)-Clta*, which led to elevated fluorescence at the blastocyst stage, demonstrating functionality of the GOGREEN system *in vivo* using epifluorescence and an exogenous *mNG2(11)*-tagged protein.

### Fluorescence reconstitution by split fluorescent protein knock-in

**Fig. 3.**
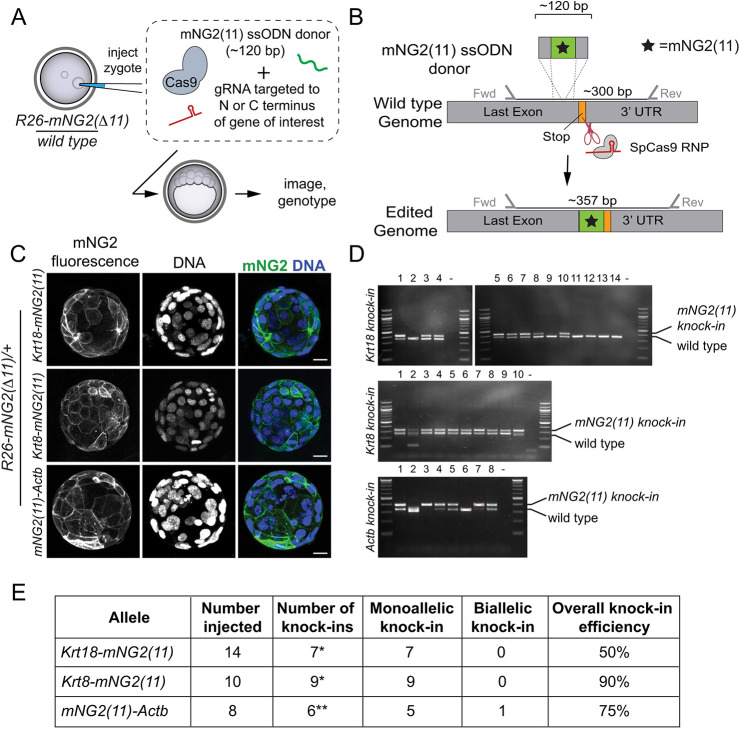
**The GOGREEN system enables detection of endogenous proteins.** (A) Experimental design: *R26-mGN2(Δ11)/+* knock-in zygotes (generated per cross shown in Fig. 2A) are injected with CRISPR/Cas9 targeting reagents to knock *mNG2(11)* into loci of interest, in frame with target proteins. Embryos are then cultured *ex vivo*, imaged and genotyped to evaluate the efficiency of *mNG2(11)* knock-in. gRNA, guide RNA; ssODN, single-stranded oligodeoxyribonucleotide. (B) Overview of strategy for targeting *mNG2(11)* to genomic loci to produce fusion proteins. (C) The GOGREEN system enables detection of endogenous cytoskeletal proteins including intermediate filaments KRT18, KRT8 and ACTB. Note that 100% of embryos inherited *R26-mNG2(Δ11)*. Scale bars: 20 µm. Sample sizes in E. (D) PCR genotyping of embryos, including those shown in C, to identify which embryos were successful *mNG2(11)* knock-ins. (E) Summary of *mNG2(11)* knock-in results observed for the three loci shown. *, one additional embryo presented the knock-in genotype, but not the expected fluorescent phenotype; **, one additional embryo presented the fluorescent phenotype, but not the expected genotype.

We next aimed to evaluate the performance of the GOGREEN system when *mNG2(11)* was endogenously expressed from several genomic loci. Our goal was to derive *R26-mNG2(Δ11)*/+ zygotes and, in these, perform CRISPR/Cas9-mediated knock-in of *mNG2(11)* in frame with proteins of interest (Fig. 3A) to produce mNG2(11) fusion proteins capable of complementing mNG2(Δ11) and reporting endogenous protein patterns.

To achieve in-frame *mNG2(11)* knock-in, we designed targeting constructs encoding the 16-amino acid mNG2(11), plus a three-amino acid linker, flanked by genomic locus-specific homology arms of 30 nucleotides each (Fig. 3B). Resulting targeting constructs ranged from 117 to 120 nucleotides in length, permitting their synthesis as a single-stranded oligodeoxyribonucleotide (ssODN) by a commercial vendor (see Materials and Methods). For our first knock-in attempts, we targeted cytoskeletal proteins, including intermediate filaments and beta-actin, because their subcellular localizations in mouse preimplantation have long been known (Chisholm and Houliston, 1987; Coonen et al., 1993; Reima and Lehtonen, 1985).

We designed CRISPR reagents to knock *mNG2(11)* in-frame with keratins 8 and 18 (*Krt8* and *Krt18*), as well as actin, beta (*Actb*)*.* Following injection of the knock-in mixture into *R26-mNG2(Δ11)*/+ zygotes, embryos were cultured to the blastocyst stage, and then imaged by confocal microscopy. For each knock-in, we observed the very unique fluorescent meshwork of cortical filamentous proteins expected, in accordance with published observations (Fig. 3C) (Coonen et al., 1993; Lim et al., 2020; Ralston and Rossant, 2008; Reima and Lehtonen, 1985). These observations are indicative of faithful protein reporting. Individual embryos were then harvested, and gene targeting was evaluated by PCR (Fig. 3D) and sequencing. In all cases, monoallelic targeting was highly efficient (Fig. 3E). These observations demonstrate the utility of the GOGREEN system for efficiently reporting the localization of endogenous proteins *in vivo*. Given that the dynamics of cytoskeletal protein localization and turnover during preimplantation development are actively studied (Anani et al., 2014; Schwarz et al., 2015; Zenker et al., 2018), the GOGREEN system could provide new tools because these proteins are usually visualized either in fixed embryos or by injection of mRNAs encoding tagged proteins, both of which could introduce unwanted artifacts.

### Fluorescence complementation in the nuclear compartment

Thus far, we had evaluated the ability of the GOGREEN system to report endogenous cytoplasmic proteins *in vivo*. However, we were uncertain whether the GOGREEN system could effectively report the dynamics of endogenous nuclear protein expression, owing to the possibility that the two components of the GOGREEN system might end up separated by the nuclear membrane.

**Fig. 4.**
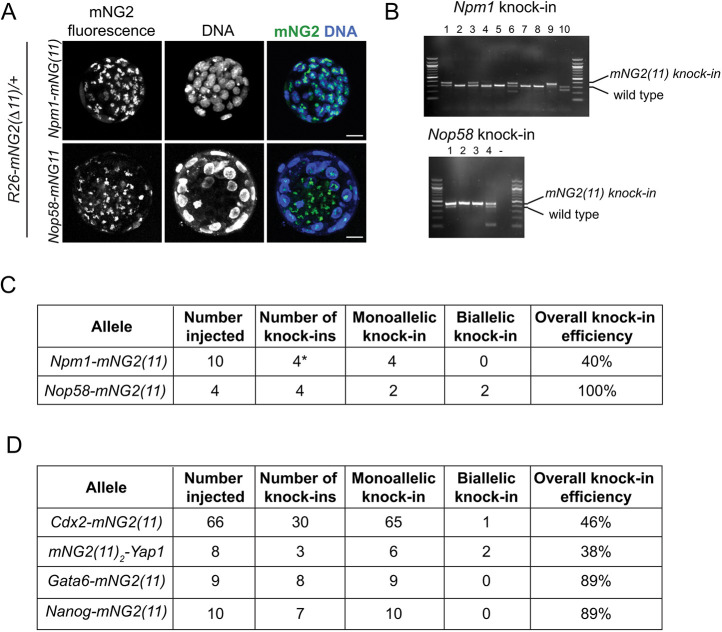
**The GOGREEN system can report endogenous protein localization in the nucleus.** (A) In the *R26-mNG2(Δ11)* background, knock-in of *mNG2(11)* in frame with the coding regions of two different nuclear proteins demonstrates fluorescent reconstitution of mNG2 and localization within nuclei. Scale bars: 20 µm. Sample sizes in C and D. (B) PCR genotyping to confirm knock-in of *mNG2(11)* into indicated loci for individual embryos, including those shown in A. (C) Efficient knock-in of nuclear proteins shown in A and B. *, one additional embryo presented the fluorescent phenotype but not the expected genotype. (D) Summary of *mNG2(11)* knock-in efficiencies, as determined by PCR genotyping, that were undetectable using the GOGREEN system.

To investigate the performance of the GOGREEN system in visualizing nuclear proteins *in vivo*, we evaluated fluorescence in embryos after targeting the genes nucleophosmin (*Npm1*) and NOP58 ribonucleoprotein (*Nop58*), which both encode nucleolar proteins. As for previous experiments, we targeted *mNG2(11)* in frame with target genes in the *R26-mNG2(Δ11)*/+ genetic background. Remarkably, we were able to detect fluorescence within the nuclear compartment (Fig. 4A) in embryonic cells following *mNG2(11)* knock-in (Fig. 4B,C). *Npm1* fluorescence recapitulated the pattern reported by immunofluorescence (Vogt et al., 2012), while the observed *Nop58* pattern is novel. These observations indicate that the nuclear envelope does not necessarily present a barrier to fluorescence complementation, in spite of the fact that the GOGREEN components lack nuclear localization sequences.

### Robust detection of low-abundance endogenous proteins

Having observed that the GOGREEN system can detect both cytoplasmic and nuclear proteins, we next tested its performance in reporting transcription factor localization, because there is great interest in imaging transcription factor dynamics in living preimplantation embryos (Gu et al., 2018; McDole et al., 2011; Posfai et al., 2017; Saiz et al., 2013, 2015). We next evaluated CDX2, YAP1, GATA6 and NANOG, four transcription factors with essential activities during preimplantation development (Frum et al., 2018; Mitsui et al., 2003; Schrode et al., 2014; Strumpf et al., 2005). However, we were unable to detect appreciable fluorescent signal in embryos of any of these four *mNG2(11)* knock-ins in the *mNG2(Δ11)* background (Fig. 4D). We therefore developed a second and alternative knock-in tagging strategy for detecting endogenous transcription factors.

**Fig. 5.**
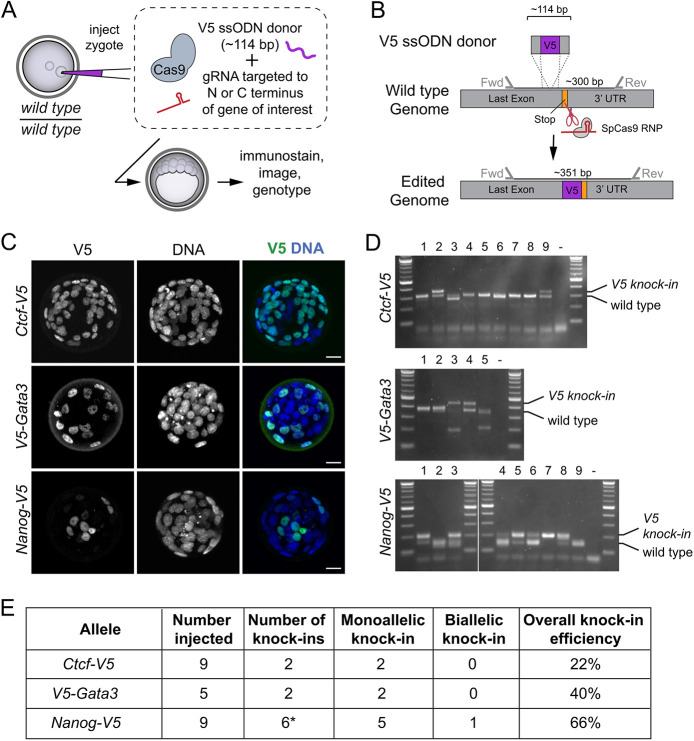
**A V5-based system for the detection of diverse transcription factors with a single antibody*.*** (A) Strategy for knocking the V5-encoding gene into loci of interest in wild-type zygotes, to enable streamlined detection of diverse endogenous proteins with a monoclonal anti-V5 antibody. (B) Overview of V5 targeting strategy. (C) Examples of proteins detected as V5 fusion proteins, following knock-in as illustrated in A and B. Scale bars: 20 µm. Sample sizes in E. (D) PCR genotyping of embryos to confirm V5 knock-in at indicated genomic loci, including those shown in C. (E) Summary of V5 knock-in efficiency at indicated loci. *, one additional embryo presented the knock-in genotype, but not the expected fluorescent phenotype.

We selected the V5 epitope, a 14-amino acid protein derived from the simian virus 5 (SV5) paramyxovirus because its small size promised high knock-in efficiency and because of the existence of low background, commercially available, monoclonal anti-V5 antibody that could be used for immunofluorescent detection of V5-tagged proteins in embryos. We then designed V5-encoding targeting constructs for generating in-frame V5 fusion proteins (Fig. 5A,B).

We targeted the V5 tag in frame with key transcription factors in zygotes, and then observed immunofluorescence patterns in blastocysts by confocal microscopy. We were able to detect V5 signals that were clear and specific after targeting the nuclear factors such as GATA3, CTCF and NANOG (Fig. 5C-E). Importantly, the patterns of V5-tagged GATA3, NANOG and CTCF recapitulated their reported expression patterns in blastocysts (Home et al., 2009; Marcho et al., 2015; Ralston et al., 2010; Strumpf et al., 2005). In a parallel set of experiments, we harvested embryos prior to blastocyst stage and then co-stained these with anti-V5 and anti-NANOG antibodies. In these embryos, we detected NANOG expression in every V5-expressing cell (Fig. S1), confirming the utility of the V5 knock-in approach for faithfully reporting gene expression, even at preimplantation stages prior to blastocyst. These observations highlight the utility of the V5-tagging system to evaluate the endogenous expression patterns of known or novel transcription factors.

**Fig. 6.**
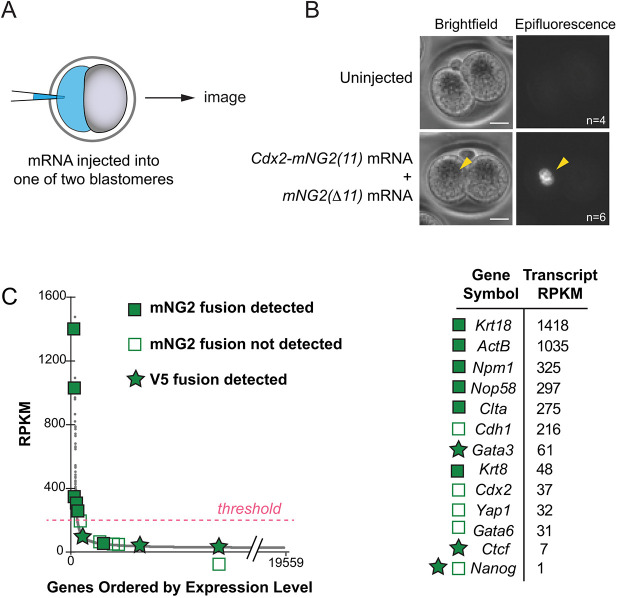
**Complementary systems enable detection of endogenous proteins over a range of expression levels.** (A) Overview of experimental design: mRNA injection into one of two blastomeres of the early mouse embryo, followed by imaging. (B) Overexpression of mRNAs encoding *mNG2(Δ11)* and *Cdx2-mNG2(11)* leads to reconstituted fluorescence in the nucleus of the injected blastomere. *n*, number of embryos presenting the phenotype shown. Scale bars: 20 µm. (C) Relative abundance of endogenous mRNAs encoding tagged proteins, as measured by RNA-seq (Aksoy et al., 2013), and detection results using GOGREEN or V5 systems. Each gray dot indicates a unique gene transcript. RPKM, reads per kilobase per million reads. For all genes shown, *mNG2(11)* or *V5* knock-in was confirmed by PCR.

Having observed that V5 outperformed the GOGREEN system, in terms of transcription factor detection, we hypothesized that protein abundance could be the limiting factor for detection using the GOGREEN system. Consistent with this hypothesis, we were able to detect the transcription factor CDX2 using GOGREEN when *Cdx2-mNG2(11)* was overexpressed by mRNA injection (Fig. 6A,B). Finally, we evaluated the abundance of transcripts encoding proteins evaluated in this study, as measured by RNA sequencing (RNA-seq) of individual blastocysts (Aksoy et al., 2013). Remarkably, transcript abundance predicted protein detectability using the GOGREEN system (Fig. 6C). Moreover, proteins of extremely low abundance could still be detected using the V5 system. This analysis therefore provides a guideline for informing subsequent experimental design and for selecting the optimal protein tagging approach. Ultimately, the GOGREEN and V5 systems together enable detection of endogenous proteins across the range of protein expression levels, facilitating multiple downstream applications and opening doors for new discoveries.

## DISCUSSION

Split GFP and V5 epitope tagging have been used for protein detection in cell lines and in some animal models (Hefel and Smolikove, 2019; Kamiyama et al., 2016; Kim et al., 2012; Leonetti et al., 2016; To et al., 2016; Yamagata and Sanes, 2012; Yang et al., 2013), but their use as knock-in mouse reporters has not been systematically compared across diverse genomic loci. Here, we presented a systematic comparison of their performance, sensitivity and efficiency of endogenous protein reporting. We note that both approaches are similarly efficient, averaging ∼60% knock-in efficiency across more than a dozen loci tested. This rate is much higher than targeting full-length fluorescent proteins by zygote injection. For example, we observed a 6% knock-in efficiency at the *R26* locus with the nearly full-length fluorescent protein mNG2(Δ11). In fact, we observed upwards of 75-100% knock-in efficiency for multiple loci, which exceeds allele inheritance rates in most mating strategies. Moreover, the relatively short length of the ssODN enables higher efficiency targeting and ease of synthesis, bypassing traditional molecular cloning methods required for producing longer donors. Finally, tagging with GOGREEN and V5 enables efficient detection of endogenous proteins, thereby circumventing artifacts caused by imaging fluorescently tagged, overexpressed proteins.

We note opportunities for applying biochemical and molecular techniques *in vivo*. V5 is commonly used for purifying proteins from cells and tissues for the downstream identification of protein or nucleotide interactions, including immunoprecipitation-western blotting or mass spectrometry, chromatin-immunoprecipitation or ribonucleotide pulldown and sequencing (ChIP-seq, RIP-seq), or cleavage under targets and release using nuclease (CUT&RUN) (Hainer et al., 2019; Skene and Henikoff, 2017). We therefore envision that the approaches described here could be used to generate stable mouse lines that enable anti-V5 antibody-mediated discovery of protein localization patterns, protein- and RNA-binding partners and DNA-binding sites throughout the genome.

Both GOGREEN and V5 systems present exciting opportunities for biological investigation outside of preimplantation mouse development as well. For example, *V5* or *mNG2(11)* knock-in embryos could be transferred to recipient females to allow for postimplantation development so that protein localization can be evaluated in later developmental processes or in adult tissues and organs. Additionally, both the GOGREEN and V5 systems could be adaptable to viral transduction (Yoon et al., 2018), which could extend applications to adult organs and tissues. Our studies thus provide guidelines, molecular reagents and genotyping assays to enable these applications.

In considering endogenous protein tagging applications, we identify several key considerations. First, care should be given to the design of the tagged protein, and whether the location and nature of the tag interfere with protein function. Validation for protein function and localization can be confirmed using appropriate strategies, including mouse genetics and, if possible, by confirming protein localization by immunofluorescence. Second, guide RNA (gRNA) design should follow best practices so as to minimize the chance of on/off-target indel alleles; targeting protein C-termini may help avoid unwanted phenotypes caused by frame-shift mutations. Third, the genotyping strategy should confirm that the tag has been knocked in in frame with the target protein at the sequence level. Related to this, strategies for identifying random ssODN insertions should be considered (Lanza et al., 2018). Finally, if microinjection is to be used as the delivery method, consultation with institutional transgenic facility with proper technical expertise should be sought, when available, to ensure optimal experimental design.

Finally, both the GOGREEN and V5 systems could also be used in embryos from species such as humans or other primates, for which breeding to establish knock-in lines is either inappropriate or impractical. There would be additional advantages to applying either system to emerging mammalian models, such as marsupials, for which protein-specific antibodies have not yet been developed. For live imaging, *mNG2(Δ11)* could be provided by mRNA injection, while *mNG2(11)* would be knocked in frame into genes of interest. If fixed imaging of low abundance proteins is preferred, then V5 could be knocked in. Either system promises new opportunities for the discovery of developmental principles in mouse as well as understudied mammalian species.

## MATERIALS AND METHODS

### Experimental design

#### Animal use

All animal research was conducted in accordance with the guidelines and approval of the Michigan State University (MSU) Institutional Animal Care and Use Committee. Most experiments were performed using male or female CD-1 mice, at least 6-8 weeks of age, maintained on a 12-h day/night cycle with food and water *ad libitum*.

#### Plasmid construction

The pR26-CAG-mNG2(Δ11) targeting vector was cloned by insertion of a synthesized dsDNA fragment encoding mNG2(Δ11) (Table S4) into the previously published vector pR26-AsisI/MluI (Addgene #74286) (Chu et al., 2016) via restriction/ligation with AsisI and MluI. After cloning, the Lox-Stop-Lox site was removed by exposure to recombinant Cre recombinase (NEB), using the NEB standard protocol. The *in vitro* transcription plasmids for *Clta-mNG2(11)*, *mNG2(Δ11)* and *Cdx2-mNG2(11)* were cloned by inserting a synthesized dsDNA fragment (Table S4) containing the respective coding sequence into a pcDNA3.1-poly(A)83 vector (Yamagata et al., 2005) downstream of the T7 promoter via restriction ligation with HindIII and NotI. pX459-sgR26-1 was generated by inserting the gRNA sequence targeting *R26* (Table S3) into pSpCas9(BB)-2A-Puro (PX459) V2.0 (Addgene #62988) via restriction ligation with BbsI.

#### mRNA synthesis

*In vitro* transcription (IVT) was performed using a T7 mMessage mMachine kit (Life Technologies). Each IVT construct was digested with XbaI, followed by ethanol precipitation, and was then used in the IVT reaction as per the manufacturer's instructions. Resulting mRNA was purified using a MEGAclear kit (Ambion). mRNA quantity and quality were assessed by a Nanodrop spectrophotometer and by agarose gel, respectively.

#### Zygote and two-cell embryo microinjection

Target-specific crispr RNA (crRNA) and non-variable trans-activating crispr RNA (tracrRNA) obtained from Integrated DNA Technologies (IDT) were each suspended in injection buffer (1 mM Tris-HCl, pH 7.5; 0.1 mM EDTA), mixed at a 1:1 molar ratio and annealed in a thermal cycler by ramping down from 95°C to 25°C at 0.1°C/s. The annealed RNAs were then mixed with recombinant Cas9 protein at a 5:1 RNA:Cas9 molar ratio and allowed to form ribonucleoproteins (RNPs) for 15 min at room temperature. RNPs were mixed with donor ssODN synthesized as Ultramers from IDT (Table S2) and diluted to working concentrations (Table S5) by addition of injection buffer. For mRNA injections, each mRNA was diluted in injection buffer to 350 ng/µl and injected either into one pronucleus of the mouse zygote or into one blastomere of the two-cell mouse embryo. Injection mixes and mRNAs were aliquoted and stored at −80°C, avoiding freeze/thaw cycles.

Zygotes were harvested from naturally mated pregnant mice on the day that copulatory plugs were detected. Oviducts were flushed with M2 medium (Millipore Sigma), and then injection mix was delivered into one pronucleus or the nucleus of one blastomere via microinjection (Nagy et al., 2003). Injected zygotes were cultured in KSOM+amino acids (AA) (Millipore Sigma) for up to 5 days before being fixed or imaged live. Only embryos that survived injection and appeared to have cavitated or to be attempting cavitation were included in the analysis.

#### Generation of *R26-mNG2(Δ11)* mouse line

The *R26-mNG2(Δ11)* mouse line was generated by zygote microinjection at the MSU Transgenic and Genome Editing Facility. *R26* sgRNA was synthesized by IVT of a PCR-amplified region of pX459-sgR26-1 using the Life Technologies MEGAshortscript T7 kit. Transcripts were subsequently purified using the MEGAclear kit. A mixture containing 5 ng/µl circular pR26-CAG-mNG(Δ11) and 125 ng/µl Cas9 RNP complexed with *sgR26* gRNA was injected into one pronucleus of C57BL/6J mouse zygotes. Zygotes were then transferred to CD-1 recipient mice. After birth, tail tips were screened by PCR for successful integration of the *R26-CAG-mNG(Δ11)* allele.

#### Immunofluorescence and confocal microscopy

V5 was detected by mouse anti-V5 antibody (Thermo Fisher Scientific, R96025). Embryos were fixed with 4% formaldehyde (Polysciences) for 10 min, permeabilized with 0.5% Triton X-100 (Millipore Sigma) for 30 min, and then blocked in 10% fetal bovine serum (Hyclone) with 0.1% Triton X-100 for 1 h at room temperature. Embryos were then incubated in anti-V5 antibody at a dilution of 1:400 in blocking solution at 4°C overnight. The next day, embryos were stained with goat anti-mouse Alexa488 (Invitrogen, A-11030) at a 1:400 dilution in blocking solution for 1 h at room temperature. Embryos were then stained for 10 min at room temperature in 50 µM Hoechst nucleic acid stain (Thermo Fisher Scientific) or DRAQ5 (Cell Signaling Technology, 4084S; 1:400 dilution). Rabbit-anti-NANOG (Reprocell, RCAB002P-F) was used at 1:400 dilution, with Cy3-conjugated donkey-anti-rabbit IgG (Jackson ImmunoResearch, 711-165-152) at 1:400 dilution. Split mNG2 embryos were imaged either fixed or live after Hoechst staining. Imaging was performed using an Olympus FluoView FV1000 Confocal Laser Scanning Microscope system with a 20× UPlanFLN objective (0.5 NA) and 5× digital zoom or with a 60× PlanApoN oil (NA 1.42) objective. For each embryo, *z*-stacks were collected with 3 µm intervals between optical sections. Optical sections are displayed as an intensity projection over the *z*-axis. Figures were prepared using FIJI, Adobe Photoshop and Adobe Illustrator.

#### Genotyping

To genotype embryos, genomic DNA was extracted from single blastocysts by placing each blastocyst in a microtube containing 4.4 µl extraction buffer (REDExtract-N-Amp Tissue PCR Kit, Millipore Sigma) mixed with 1.1 µl tissue prep buffer, and then incubating tubes at 56°C for 30 min, 24°C for 5 min and 95°C for 5 min. After incubation, 5 µl neutralization buffer was added to each tube. In subsequent reactions, 1 µl of embryo extract was used as a PCR template, and locus-specific primers (Table S1). To genotype adult mice, genomic DNA was extracted from ear punch biopsies using a Wizard SV Genomic DNA Purification System (Promega), and PCR was performed using Herculase II Polymerase (Agilent).

#### Sequencing

To confirm the identity of select PCR products, the products were directly cloned into pCR2.1 TOPO using an Invitrogen TOPO TA Cloning Kit (Invitrogen). Plasmids containing the PCR product were prepped with the Spin Miniprep Kit (Qiagen) and then sequenced by RTSF Sanger method at the Genomics Core at MSU.

#### *mNG2(Δ11)* ESC derivation

R1/E ESCs (American Type Culture Collection) were cultured on CF-1 feeder mouse embryonic fibroblasts (MEFs; Applied Stem Cell) in ESC medium [Dulbecco's modified Eagle medium (DMEM) supplemented with 1000 U/ml leukemia inhibitory factor (Millipore Sigma), 15% (v/v) fetal bovine serum (Hyclone), 2 mM L-Glutamax (Thermo Fisher Scientific), 0.1 mM beta-mercaptoethanol (Millipore Sigma), 0.1 mM minimum essential medium (MEM) non-essential amino acids (Millipore Sigma), 1 mM sodium pyruvate (Millipore Sigma) and 1% (v/v) penicillin/streptomycin (Gibco)]. Passage 13 R1/E ESCs were cultured to ∼70% confluence in a 10 cm dish, and then electroporated with pX459-sgR26-1 and pR26-CAG-mNG2(Δ11) as follows: pelleted cells were resuspended in 800 µl Embryo-Max Electroporation Buffer (Millipore Sigma) containing 20 µg of each plasmid, and cells were then electroporated in a 0.4 cm electrode gap electroporation cuvette (Bio-Rad) using a Bio-Rad Gene Pulser XCell electoporator (250 V, 500 µF, infinite Ω). Subsequently, 400 µl electroporated cells were diluted in 10 ml ESC medium and then plated on a 10 cm dish on puromycin-resistant DR4 feeder MEFs (Applied Stem Cell). After 24 h, selection was started with ESC medium containing 1.25 µg/ml puromycin (Gibco). After 12 days, colonies were picked into 96-well plates and expanded over several more passages. Cell lines were genotyped by PCR using *R26F3* and *SAR* primers to detect insertion of *mNG2(Δ11)* in the *R26* locus (Table S1).

## Supplementary Material

Supplementary information

Reviewer comments
