# Supplementary material for: Efficient generation of endogenous protein reporters for mouse development
Source: Development. 2021 Jun 29;148(13):dev197418. doi: 10.1242/dev.197418 (PMC8276983; doi:10.1242/dev.197418)
Supplement: Supplementary information [file develop-148-197418-s1.pdf]

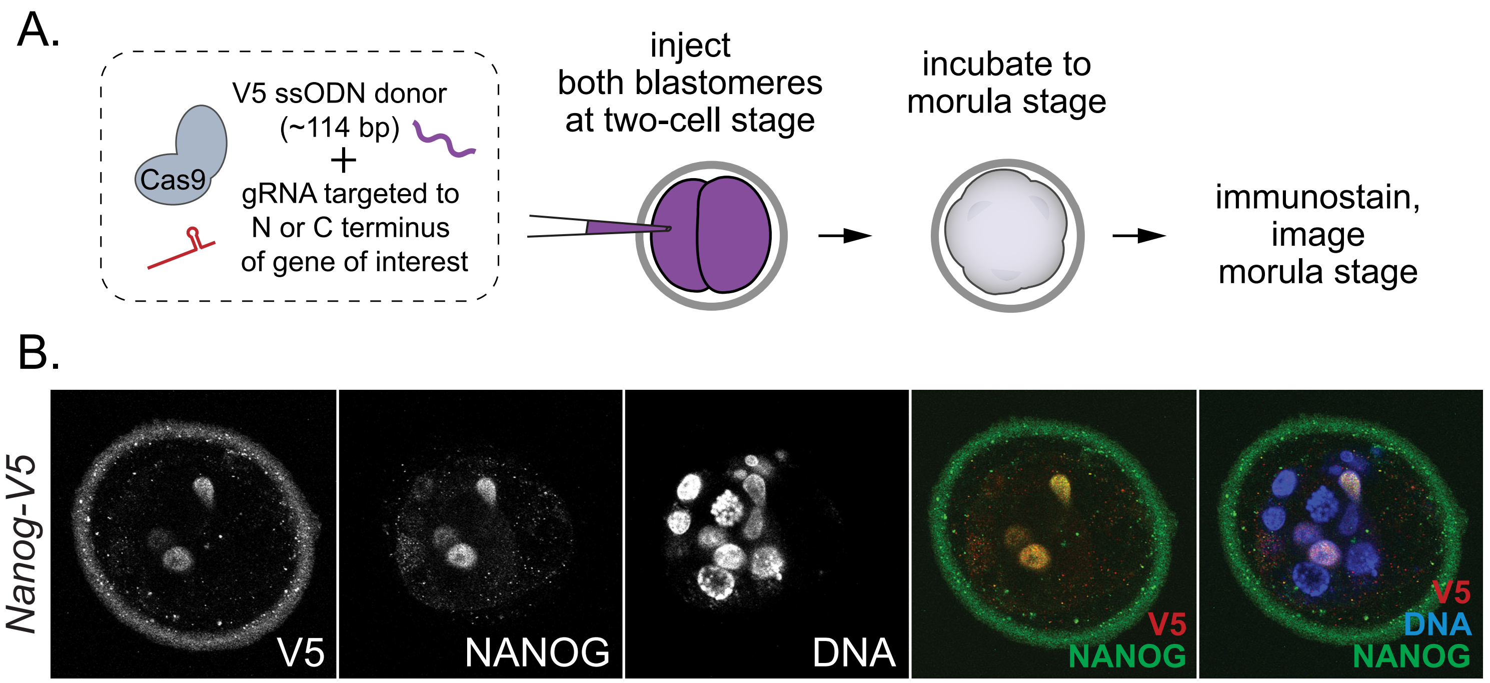

Fig. S1. NANOG-V5 is detected in NANOG-expressing cells. A) Experimental design. B) Immunofluorescent detection of V5 and NANOG in embryos prior to the blastocyst stage (n =3).

Table S1. Genotyping primers used in this study

Allele	Forward (5' to 3')	Reverse (5' to 3')
R26-mNG2(Δ11)	CTGCCCGAGCGGAAACGCCACTGAC	CCTGGACTACTGCGCCCTACAGA
Krt18-mNG2(11)	GGCTGTTTATAACTAAGGCTTGGTC	GGACAGTCATATCTCCTACTTCGTC
Krt8-mNG2(11)	TGTGGTTGTGAAGAAGATTGAAACC	ATACAACTGAATTGGGTTTGGATGG
mNG2(11)-Actb	CCAGCGTTTGCCTTTTATGGTAATA	CACTCCCAAAGTAACAGGTCACTT
Npm1-mNG2(11)	GGCAACACTGGCCATAAAGTATTTA	CAAACACAGTAGGGAAAGTTCTCAC
mNG2(11)-Nop58	GATATTTTAAGGCCGTCTCTTTCCG	CAACAACTCCATCTCACCTACCTTA
Ctcf-V5	CAGAATACAGGTGCAATTGAGAACA	CATCCTTGAAGTTTTCGTTCTCAGT
V5-Gata3	CTTTTGCTAAACTATCCCGCAAAGA	TTGCCTTGACCATCGATGTTAAAAA
Nanog-V5	CCACTAGGGAAAGCCATGCGCATTT	GGAAGAAGGAAGGAACCTGGCTTTGC
Cdx2-mNG2(11)	GAGAGGAAAATCAAGAAGAAGCAGC	GAGGAATCTCTTCTGAGGATTCTCG

Development • Supplementary information

Table S2. Synthesized ssODN sequences used in this study. Phosphorothioate bonds (indicated by *) were added during oligo synthesis to enhance oligo resistance to endogenous exonuclease degradation.

**Allele**	**Sequence 5' to 3'**
Krt18-mNG2(11)	T*TCCCAGGGGTTCCCTCCTTCTCTGCCTCACATCATATCGGTAAAGGCCTTTTGCCACTCCTTGAAGTTGAGCTCGGTGCCAGAGCCGTGCCTCAGAACTCTGGTGTCATTAGTCT*C
Krt8-mNG2(11)	G*TGTCCGAGTCTTCTGATGTCGTGTCCAAGGGCTCTGGCACCGAGCTCAACTTCAAGGAGTGGCAAAAGGCCTTTACCGATATGATGTGAATGGCCACTGAAGTCCTTGCCAGCCT*G
mNG2(11)-Actb	G*ACGACCAGCGCAGCGATATCGTCATCCATGCCACCTCCCATCATATCGGTAAAGGCCTTTTGCCACTCCTTGAAGTTGAGCTCGGTCATGGCGAACTATCAAGACACAAAAGAAGGCT*A
Npm1-mNG2(11)	C*AAGATCTCTGGCAGTGGAGGAAATCTCTTGGCTCTGGCACCGAGCTCAACTTCAAGGAGTGGCAAAAGGCCTTTACCGATATGATGTAAGAAAAGGGTTTAAACAGTTTGAAATA*T
mNG2(11)-Nop58	C*GCGTAGCGCCGCCCTGACCTGGTCTCATCATGACCGAGCTCAACTTCAAGGAGTGGCAAAAGGCCTTTACCGATATGATGGGAGGTGGCATGTTGGTCCTGTTTGAAACGTCCGTTGG*C
Ctcf-V5	C*CTGAGATGATCCTCAGCATGATGGACCGGGGCTCTGGCGGCAAGCCGATCCCTAACCCTCTGCTGGGCCTGGACAGCACTTGATGCTGGGGCCTTGCTCGGCACCAGGA*C
V5-Gata3	G*GGCGAGAGGGCGCGAGCACAGCCGAGGACATGGGCAAGCCGATCCCTAACCCTCTGCTGGGCCTGGACAGCACTGGAGGTGGCATGGAGGTGACTGCGGACCAGCCGCGCTG*G
Nanog-V5	A*CTTTAAGCCCAGATGTTGCGTAAGTCTCAAGTGCTGTCCAGGCCCAGCAGAGGGTTAGGGATCGGCTTGCCGCCAGAGCCTATTTCACCTGGTGGAGTCACAGAGTAGT*T
Cdx2-mNG2(11)	C*GCCGCCGCTTCAGACCACGGGAGGGGTCACATCATATCGGTAAAGGCCTTTTGCCACTCCTTGAAGTTGAGCTCGGTGCCAGAGCCCTGGGTGACAGTGGAGTTTAAAACCCCTC*C

Development • Supplementary information

Table S3. CRISPR Guides used in this study. Underlined sequence = Protospacer Adjacent Motif (PAM)

**Allele**	**Guide Sequence (5' to 3')**
R26-mNG(Δ11)	ACTCCAGTCTTTCTAGAAGATGG
Krt18-mNG2(11)	ACCAGAGTTCTGAGGCACTGAGG
Krt8-mNG2(11)	TGATGTCGTGTCCAAGTGAATGG
mNG2(11)-Actb	TGTGTCTTGATAGTTCGCCATGG
Npm1-mNG2(11)	GAGGAAATCTCTTTAAGAAAAGG
mNG2(11)-Nop58	CTGACCTGGTCTCATCATGTTGG
Ctcf-V5	GAGCAAGGCCCCAGCATCACCGG
V5-Gata3	GAGCACAGCCGAGGACATGGAGG
Nanog-V5	CGTAAGTCTCATATTTCACCTGG
Cdx2-mNG2(11)	CAGACCACGGGAGGGGTCACTGG

Table S4. dsDNA fragments synthesized for this study.

Click here to download Table S4

Table S5: Concentrations of Cas9 RNP and ssODN used for targeting each gene.

Gene Targeted	Tag	Cas9 RNP (ng/µl)	ssODN (ng/µl)
Krt8	mNG2(11)	100	20
Actb	mNG2(11)	100	20
Krt18	mNG2(11)	100	20
Nop58	mNG2(11)	100	20
Npm1	mNG2(11)	100	10
Ctcf	V5	25	5
Gata3	V5	100	20
Nanog	V5	100	20
