# Supplementary material for: Efficient generation of endogenous protein reporters for mouse development
Source: Development. 2021 Jun 29;148(13):dev197418. doi: 10.1242/dev.197418 (PMC8276983; doi:10.1242/dev.197418)
Supplement: Reviewer comments [file develop-148-197418_review_history.pdf]

Efficient generation of endogenous protein reporters for mouse development

Daniel O'Hagan, Robin E. Kruger, Bin Gu and Amy Ralston
DOI: 10.1242/dev.197418

Editor: Liz Robertson

Review timeline
Original submission: 28 September 2020
Editorial decision: 26 October 2020
First revision received: 12 March 2021
Editorial decision: 30 March 2021
Second revision received: 19 May 2021
Accepted: 20 May 2021

Original submission

<u>First decision letter</u>

MS ID#: DEVELOP/2020/197418

MS TITLE: Efficient generation of endogenous protein reporters for mouse preimplantation embryos

AUTHORS: Amy Ralston and Daniel O'Hagan

I have now received all the referees' reports on the above manuscript, and have reached a decision. The referees' comments are appended below, or you can access them online: please go to BenchPress and click on the 'Manuscripts with Decisions' queue in the Author Area.

As you will see, the referees express considerable interest in your work, but have some criticisms and recommend a revision of your manuscript before we can consider publication. If you are able to revise the manuscript along the lines suggested, which may involve further experiments, I will be happy receive a revised version of the manuscript. Your revised paper will be re-reviewed by one or more of the original referees, and acceptance of your manuscript will depend on your addressing satisfactorily the reviewers' major concerns. Please also note that Development will normally permit only one round of major revision.

We are aware that you may be experiencing disruption to the normal running of your lab that make experimental revisions challenging. If it would be helpful, we encourage you to contact us to discuss your revision in greater detail. Please send us a point-by-point response indicating where you are able to address concerns raised (either experimentally or by changes to the text) and where you will not be able to do so within the normal timeframe of a revision. We will then provide further guidance. Please also note that we are happy to extend revision timeframes as necessary.

Please attend to all of the reviewers' comments and ensure that you clearly highlight all changes made in the revised manuscript. Please avoid using 'Tracked changes' in Word files as these are lost in PDF conversion. I should be grateful if you would also provide a point-by-point response detailing how you have dealt with the points raised by the reviewers in the 'Response to Reviewers' box. If you do not agree with any of their criticisms or suggestions please explain clearly why this is so.

© 2021. Published by The Company of Biologists under the terms of the Creative Commons Attribution License (https://creativecommons.org/licenses/by/4.0/).

Reviewer 1

Advance summary and potential significance to field

In their manuscript O'Hagan and Ralston present two new strategies for high throughput production of endogenous protein reporters in mouse embryos. For high abundance proteins the authors propose the use of a split fluorescent protein mNeonGreen2, where the bigger unit (mNG2(D11) is either delivered by mRNA injection or via establishment of a mouse line capable of constitutive expression of mNG2(D11) and the smaller unit (mNG2(11) can be endogenously expressed from a variety of genomic loci. The authors called this system GOGREEN. For low abundance proteins (like some of the transcription factors) the authors propose an alternative approach. They selected the V5 epitope, a 14 amino acid protein derived from the simian virus 5 (SV5) due to its high knock-in efficiency and the fact that V5-tagged proteins can be later detected with low background, commercially available, monoclonal anti-V5 antibody.
O'Hagan and Ralston argue that the GOGREEN system could provide new tools to study the protein localisation in live embryos, while avoiding the problem caused by the injection of mRNAs encoding tagged proteins that could introduce unwanted artefacts (like the effects of overexpression). The authors provided compelling evidence that both approaches can be successfully used to detect various endogenous proteins at least at the blastocyst stage (and in one case at the 2 cells stage).

Comments for the author

Although both techniques offer an interesting alternative for antibody staining (especially for the species that commercially available antibodies may not yet exist), however several controls need to be provided first before both techniques can be presented as fateful representations of the endogenous protein's distribution (see below).
Moreover, I would like to point out that both techniques are nowhere near as easy to perform as classical antibody staining. Not every lab will be able to perform microinjection due to the lack of equipment or necessary skills. While the authors suggested that it can be delivered by other means, it is not clear how successfully this can be performed on preimplantation embryos. As for the use of mice lines, considering that the cost of maintaining the colony of mNG2(delta11) mice can be substantial, I am not sure if the argument that this is a cheaper option than antibody staining is particularly valid.
Having said that, I can see clear benefits for the use of both methods for the detection of the endogenous proteins in live embryos (especially non-rodent mammals).

Major points to consider

- It is not clear how well the new system would perform if compared to the commercially available antibodies. Therefore, the authors should provide side-by-side comparisons of V5-based system signal together with antibody staining for the protein of interest (at least two different examples). Similar observations should be provided for the GOGREEN system. For example, one can imagine that in some cases
(especially when not much of the endogenous protein of interest is present at certain stages and/or in certain cells) the GOGREEN system will miss these cells. At the moment it is not clear to me whether the V5-based system is able to detect low levels of transcription factors that can be visualized with a good commercial antibody.
- Images of embryos from one or two additional developmental stages should be provided (together with antibody staining) in order to prove that the strength of the signal at the earlier stages allows for protein detection. Most of the proteins will be produced on much higher levels at the blastocyst stage, therefore investigation of the earlier stages is crucial to provide the evidence that both systems can be used for the whole preimplantation development. Providing such stage by stage images of the same embryo would strengthen the authors claim that both systems can be used during the whole preimplantation period.
- I believe that providing short time lapse videos that visualize endogenous proteins in developing embryos would greatly strengthen the manuscript but I appreciate that this may not be easy to do.

Minor point
Figure 2B – levels of background are very different between epifluorescence images therefore it is difficult to say how strong is the signal on each image.

© 2021. Published by The Company of Biologists under the terms of the Creative Commons Attribution License (https://creativecommons.org/licenses/by/4.0/).

Reviewer 2

Advance summary and potential significance to field

In this Techniques and Resources manuscripts, the authors provide detailed methods to endogenously tag proteins of interest with high knock-in efficiency. This is an important addition to the repertoire of molecular techniques that can be used to study endogenous protein expression dynamics. This method will enable the investigation of protein expression dynamics of factors where there are currently no available antibodies or tagging endogenous genes in species where proteins expression dynamics have not been evaluated by live embryo imaging.

Embryos constitutively expressing mNG2(delta 11) were co-injected with an RNP targeting endogenous genes together with a synthetic ssODN that encoded the complementary mNG2(11) sequence, a linker sequence and short homology arms. ssODNs are known to have higher knock-in efficiency and can be introduced into zygotes, which is an advantage over other CRISPR-Cas9 targeting methods. The authors demonstrated efficient targeting and the study of fluorescent protein expression dynamics using a number of examples showing the robustness of the technique.

In the other approach a V5 tag was introduced into the endogenous locus and IF was performed using a V5 antibody. The authors showed the utility of this approach in faithfully recapitulating the known expression pattern of a number of transcription factors as well as CTCF, whose expression has not been previously characterised in early embryos. This will therefore be a very useful approach for tagging proteins where there are not currently working antibodies to investigate endogenous protein expression. The authors also showed that transcript abundance could be used as a predictor of which of the novel methods described in the manuscript could be used for protein tagging. In all, I think this is an important methodological advance, which will be useful for the community.

Comments for the author

I have a few points the authors should consider:

1. I wondered if the authors considered the frequency with which they generate indel mutations at the on-target site compared to ssODN-mediated incorporation of the mNG2(11) or V5 tag? Similarly, I think there may be an interest in the frequency of mono- versus bi-allelic targeting. The genomic DNA exists for targeted embryos and this could be used to investigate, by target amplicon MiSeq analysis, the genotype of the embryos and whether they harbour any mutations. Alternatively, the on-target PCR amplicon could be used in TA cloning – here the authors may need to evaluate a number of clones given the likelihood of mosaicism which is why I think MiSeq may be slightly easier and a more robust way to evaluate the genotype.

2. Could the authors discuss in the Discussion section the likelihood of the ssODN integrating elsewhere in the genome (not at the gRNA on-target site)? Is it known by Southern blot, or other methods, whether (given the shortness of the homology arms) these fragments may integrate elsewhere? If it is not known I think it may be helpful for the authors to comment on this possibility in the discussion section and ways that this could be evaluated in the future.

3. For consistency it would be helpful if all of the schematics were more uniform, for example between Figure 1B and Figure 3B. In one, the sgRNA sequence is shown and in the other there is a schematic of an RNP without the sequence information. Streamlining this information will make the figures easier to understand.

Minor suggestions:

1. There are a number of typos, for example Line 34 should be: from preimplantation embryos

2. Line 42 should be during preimplantation development. It may also be helpful to add that this has conventionally been achieved through homologous recombination.

3. Line 44 should be: even when CRISPR/Cas9 mediated genome editing is used

© 2021. Published by The Company of Biologists under the terms of the Creative Commons Attribution License (https://creativecommons.org/licenses/by/4.0/).

4. On lines 45-46 it would be helpful to point out that while antibody-based approaches are informative to investigate protein expression at static time points, these approaches lack the temporal resolution and expression dynamics that can be achieved by live cell imaging. This may be especially important for factors that undergo rapid changes in expression between cell types.

5. It would be a nice complement to the author's unique tagging methods if they could provide immunofluorescence images, if they already have these, to include in the manuscript. Comparing the IF expression of some of the factors using antibodies against say the Npm1 or KRT18 to the tagged proteins to investigate overlapping expression would be an informative way to show that the tagging faithfully recapitulates the expected pattern of expression, especially for people not use to examining the expression of these specific proteins. I don't think this is required and is only a suggestion.

Reviewer 3

Advance summary and potential significance to field

In this manuscript O'Hagan and Ralston describe two strategies for rapid tagging and localization of endogenous proteins in the early mouse embryo. The first of these uses the split fluorescent protein mNeon Green (mNG2) to allow tagging and subsequent localization of endogenous proteins in living embryos, and the second uses the V5 epitope, which permits more sensitive localization of endogenous proteins in fixed embryos. They use these two systems to localize a number of proteins with known distributions as proof of principle, and they characterize the sensitivity of each of these systems based on subcellular localization and mRNA copy number. While the tagging methods that they use are not novel, having been previously used in cultured cell models, the particular application to mouse embryos provides a very significant advance in researchers' ability to target and visualize proteins in the mouse embryo. As the authors point out, it has enormous potential not only for analyzing mouse development, but also for analyzing development in embryos of species that aren't amenable to the conventional approaches to manipulating genes and proteins (human, marsupial). It is likely that the mNG2(delta11) mouse line that they have created will become an important tool for many labs in the future.

Comments for the author

The authors provide good data that verify the efficiency of their system and support their interpretations. It is really appealing to see the very high efficiency of successful knock-in for both the mNG2(11) and V5 sequences.

An important issue that is not addressed, however, is validation of the function of the targeted proteins, and the parameters to consider for the non-covalent binding of the two parts of the mNG2 system. There is also no discussion of the reasoning behind the choice of location for insertion of the mNG2(11) sequence.

If there are important functional domains on the carboxy terminus of the protein does that impair either the function of the tagged protein, or of the non-covalent interactions of the mNG2 components? Can the mNG2(11) sequence be inserted in other regions of the gene and successfully complement the mNG2(delta11)?

A minor issue is the quality of the fluorescence images in figure 2 - it would be very helpful to have higher resolution images, or a max projection of a Z-stack to better see the clathrin localization. Another minor issue is the terminology describing Figure 2 in the text (p. 4 lines 94-102). Clta should be defined the first time it is used (line 96). In lines 98-99, both of the injected mRNAs are described as "(delta11)", and as such should not be a positive control – I think the second should be "(11)". The result that the fluorescence for the R26-mNG2(delta11)/+ embryos injected with Clta-mNG2(11) is less than the positive control should be included in the Results section and not just in the figure legend.

© 2021. Published by The Company of Biologists under the terms of the Creative Commons Attribution License (https://creativecommons.org/licenses/by/4.0/).

First revision

Author response to reviewers' comments

Reviewer 1 Advance Summary and Potential Significance to Field:

In their manuscript O'Hagan and Ralston present two new strategies for high throughput production of endogenous protein reporters in mouse embryos. For high abundance proteins the authors propose the use of a split fluorescent protein mNeonGreen2, where the bigger unit (mNG2(D11) is either delivered by mRNA injection or via establishment of a mouse line capable of constitutive expression of mNG2(D11) and the smaller unit (mNG2(11) can be endogenously expressed from a variety of genomic loci. The authors called this system GOGREEN. For low abundance proteins (like some of the transcription factors) the authors propose an alternative approach. They selected the V5 epitope, a 14 amino acid protein derived from the simian virus 5 (SV5) due to its high knock-in efficiency and the fact that V5-tagged proteins can be later detected with low background, commercially available, monoclonal anti-V5 antibody. O'Hagan and Ralston argue that the GOGREEN system could provide new tools to study the protein localisation in live embryos, while avoiding the problem caused by the injection of mRNAs encoding tagged proteins that could introduce unwanted artefacts (like the effects of overexpression). The authors provided compelling evidence that both approaches can be successfully used to detect various endogenous proteins at least at the blastocyst stage (and in one case at the 2 cells stage).

Reviewer 1 Comments for the Author:
Although both techniques offer an interesting alternative for antibody staining (especially for the species that commercially available antibodies may not yet exist), however several controls need to be provided first before both techniques can be presented as fateful representations of the endogenous protein's distribution (see below).

Moreover, I would like to point out that both techniques are nowhere near as easy to perform as classical antibody staining. Not every lab will be able to perform microinjection due to the lack of equipment or necessary skills. While the authors suggested that it can be delivered by other means, it is not clear how successfully this can be performed on preimplantation embryos. As for the use of mice lines, considering that the cost of maintaining the colony of mNG2(delta11) mice can be substantial, I am not sure if the argument that this is a cheaper option than antibody staining is particularly valid. Having said that, I can see clear benefits for the use of both methods for the detection of the endogenous proteins in live embryos (especially non- rodent mammals).

Very much agreed. To this particular point, we have removed discussion of cost, and clarified technical expertise required (Lines 38-47 and 238-240).

Major points to consider
It is not clear how well the new system would perform if compared to the commercially available antibodies. Therefore, the authors should provide side-by-side comparisons of V5- based system signal together with antibody staining for the protein of interest (at least two different examples). Similar observations should be provided for the GOGREEN system. For example, one can imagine that in some cases (especially when not much of the endogenous protein of interest is present at certain stages and/or in certain cells) the GOGREEN system will miss these cells. At the moment it is not clear to me whether the V5-based system is able to detect low levels of transcription factors that can be visualized with a good commercial antibody.

We agree that co-staining would be required in order to draw conclusions about molecular-level detail of protein localization. Our goal here was to focus on gene expression at the cellular/embryonic level.

Accordingly, we did not prioritize co-staining with antibodies against the targeted proteins because we aimed to determine what proportion of targeted proteins would recapitulate their known expression patterns within the blastocyst lineages, such as trophectoderm and inner cell mass.

© 2021. Published by The Company of Biologists under the terms of the Creative Commons Attribution License (https://creativecommons.org/licenses/by/4.0/).

Because we observed lineage-specific expression of several markers, we concluded that the tagged proteins recapitulated known expression patterns, analysis of lineage markers.

Thus, we have taken this opportunity to incorporate the reviewer's point, in terms of which conclusions can and cannot be fairly draw from the data shown (Lines 231-233).

Images of embryos from one or two additional developmental stages should be provided (together with antibody staining) in order to prove that the strength of the signal at the earlier stages allows for protein detection. Most of the proteins will be produced on much higher levels at the blastocyst stage, therefore investigation of the earlier stages is crucial to provide the evidence that both systems can be used for the whole preimplantation development. Providing such stage by stage images of the same embryo would strengthen the authors claim that both systems can be used during the whole preimplantation period.

This is a good point. We did perform some analyses prior to blastocyst stage, and we were able to detect tagged proteins in many cases. However, our analyses were not as detailed, comprehensive, or thorough as for our blastocyst end-point studies. Unfortunately, the first author of the manuscript left the lab during the pandemic, and so we are not able to provide new data.

We do, however, feel that by demonstrating detection of low abundance transcription factors at the blastocyst stage, that we have addressed this issue indirectly. For example, at the blastocyst stage, Nanog levels are 10 RPKM, which is lower than the levels of other lineage markers (e.g., Krt8, Krt18, Cdx2, etc) at stages prior to blastocyst.

In the revised manuscript, we have changed the abstract and introduction so that the manuscript is now more clearly focused on blastocysts as a model for development.

I believe that providing short time lapse videos that visualize endogenous proteins in developing embryos would greatly strengthen the manuscript but I appreciate that this may not be easy to do.

We thank the reviewer for putting it this way. While we were in the process of undertaking live imaging experiments, we were disrupted by the pandemic, which has unfortunately forced us put this goal on hold for the time being.

Minor point

Figure 2B – levels of background are very different between epifluorescence images therefore it is difficult to say how strong is the signal on each image.

Thank you for catching that! We have replaced the original images, which had been included in error. The replacement images represent equivalent imaging conditions and are consistent with our original conclusions.

Reviewer 2 Advance Summary and Potential Significance to Field:
In this Techniques and Resources manuscripts, the authors provide detailed methods to endogenously tag proteins of interest with high knock-in efficiency. This is an important addition to the repertoire of molecular techniques that can be used to study endogenous protein expression dynamics. This method will enable the investigation of protein expression dynamics of factors where there are currently no available antibodies or tagging endogenous genes in species where proteins expression dynamics have not been evaluated by live embryo imaging.

Embryos constitutively expressing mNG2(delta 11) were co-injected with an RNP targeting endogenous genes together with a synthetic ssODN that encoded the complementary mNG2(11) sequence, a linker sequence and short homology arms. ssODNs are known to have higher knock-in efficiency and can be introduced into zygotes, which is an advantage over other CRISPR-Cas9 targeting methods. The authors demonstrated efficient targeting and the study of fluorescent protein expression dynamics using a number of examples showing the robustness of the technique.

In the other approach a V5 tag was introduced into the endogenous locus and IF was performed using a V5 antibody. The authors showed the utility of this approach in faithfully recapitulating the

© 2021. Published by The Company of Biologists under the terms of the Creative Commons Attribution License (https://creativecommons.org/licenses/by/4.0/).

known expression pattern of a number of transcription factors as well as CTCF, whose expression has not been previously characterised in early embryos. This will therefore be a very useful approach for tagging proteins where there are not currently working antibodies to investigate endogenous protein expression. The authors also showed that transcript abundance could be used as a predictor of which of the novel methods described in the manuscript could be used for protein tagging. In all, I think this is an important methodological advance, which will be useful for the community.

Reviewer 2 Comments for the Author:

I have a few points the authors should consider:

1. I wondered if the authors considered the frequency with which they generate indel mutations at the on-target site compared to ssODN-mediated incorporation of the mNG2(11) or V5 tag? Similarly, I think there may be an interest in the frequency of mono- versus bi-allelic targeting. The genomic DNA exists for targeted embryos and this could be used to investigate, by target amplicon MiSeq analysis, the genotype of the embryos and whether they harbour any mutations. Alternatively, the on-target PCR amplicon could be used in TA cloning – here the authors may need to evaluate a number of clones given the likelihood of mosaicism which is why I think MiSeq may be slightly easier and a more robust way to evaluate the genotype.

We did consider that our double-stranded DNA repair could lead to mutagenesis. For this reason, we generally targeted our tags to the C-terminus. We now discuss this consideration in the revised manuscript (Lines 233-235).

Thank you for the suggestion to evaluate mono- versus bi-allelic targeting efficiencies. We were able to determine genotypes by PCR and gel electrophoresis, and we now include these new data in the tables shown in Fig. 3E, 4C, 4D, and 5E. In general, monoallelic knock-in was more common than biallelic knock-in. This is now highlighted on Lines 125-126.

2. Could the authors discuss in the Discussion section the likelihood of the ssODN integrating elsewhere in the genome (not at the gRNA on-target site)? Is it known by Southern blot, or other methods, whether (given the shortness of the homology arms) these fragments may integrate elsewhere? If it is not known I think it may be helpful for the authors to comment on this possibility in the discussion section and ways that this could be evaluated in the future.

This is a great point, which we now discuss in terms of genotyping strategy on Lines 237-238.

3. For consistency it would be helpful if all of the schematics were more uniform, for example between Figure 1B and Figure 3B. In one, the sgRNA sequence is shown and in the other there is a schematic of an RNP without the sequence information. Streamlining this information will make the figures easier to understand.

Great suggestion; we have made the requested change.

Minor suggestions:
1. There are a number of typos, for example Line 34 should be: from preimplantation embryos
2. Line 42 should be during preimplantation development. It may also be helpful to add that this has conventionally been achieved through homologous recombination.
3. Line 44 should be: even when CRISPR/Cas9 mediated genome editing is used
4. On lines 45-46 it would be helpful to point out that while antibody-based approaches are informative to investigate protein expression at static time points, these approaches lack the temporal resolution and expression dynamics that can be achieved by live cell imaging. This may be especially important for factors that undergo rapid changes in expression between cell types.

Great! Changes made.

5. It would be a nice complement to the author’s unique tagging methods if they could provide

© 2021. Published by The Company of Biologists under the terms of the Creative Commons Attribution License (https://creativecommons.org/licenses/by/4.0/).

immunofluorescence images, if they already have these, to include in the manuscript. Comparing the IF expression of some of the factors using antibodies against say the Npm1 or KRT18 to the tagged proteins to investigate overlapping expression would be an informative way to show that the tagging faithfully recapitulates the expected pattern of expression, especially for people not use to examining the expression of these specific proteins. I don't think this is required and is only a suggestion.

Regrettably, we are not able to pursue the suggestion of costaining with protein-specific antibodies because of how the pandemic has affected our research program. We instead provide more detail on how our observed expression patterns recapitulate known patterns (Lines 121-124, 146-148, and 173-175).

Reviewer 3 Advance Summary and Potential Significance to Field:

In this manuscript O'Hagan and Ralston describe two strategies for rapid tagging and localization of endogenous proteins in the early mouse embryo. The first of these uses the split fluorescent protein mNeon Green (mNG2) to allow tagging and subsequent localization of endogenous proteins in living embryos, and the second uses the V5 epitope, which permits more sensitive localization of endogenous proteins in fixed embryos. They use these two systems to localize a number of proteins with known distributions as proof of principle, and they characterize the sensitivity of each of these systems based on subcellular localization and mRNA copy number. While the tagging methods that they use are not novel, having been previously used in cultured cell models, the particular application to mouse embryos provides a very significant advance in researchers' ability to target and visualize proteins in the mouse embryo. As the authors point out, it has enormous potential not only for analyzing mouse development, but also for analyzing development in embryos of species that aren't amenable to the conventional approaches to manipulating genes and proteins (human, marsupial). It is likely that the mNG2(delta11) mouse line that they have created will become an important tool for many labs in the future.

Reviewer 3 Comments for the Author:

The authors provide good data that verify the efficiency of their system and support their interpretations. It is really appealing to see the very high efficiency of successful knock-in for both the mNG2(11) and V5 sequences.

An important issue that is not addressed, however, is validation of the function of the targeted proteins, and the parameters to consider for the non-covalent binding of the two parts of the mNG2 system. There is also no discussion of the reasoning behind the choice of location for insertion of the mNG2(11) sequence. If there are important functional domains on the carboxy terminus of the protein, does that impair either the function of the tagged protein, or of the non- covalent interactions of the mNG2 components? Can the mNG2(11) sequence be inserted in other regions of the gene and successfully complement the mNG2(delta11)?

This is an interesting issue that we have not had the chance to systematically test, since most of our tags were inserted C-terminally so as to avoid indels that could disrupt the open reading frame. We now include a discussion of whether these tags could interfere with protein function (Lines 231-233), and note that this is a consideration whether full-length or split fluorescent proteins are used. We hope to perform more comprehensive assessments in the future as we begin our longer-term analyses of stable mouse lines carrying various tagged alleles.

A minor issue is the quality of the fluorescence images in figure 2 - it would be very helpful to have higher resolution images, or a max projection of a Z-stack to better see the clathrin localization.

Our intent was to include these images to highlight that the preliminary fluorescence characterization could be assessed on an epifluorescence microscope (as opposed to confocal) and because we chose not to focus overly on the subcellular localization of what is, in this case, an overexpressed protein. We now clarify the motivation and interpretation of this line of inquiry on Lines 91-100.

© 2021. Published by The Company of Biologists under the terms of the Creative Commons Attribution License (https://creativecommons.org/licenses/by/4.0/).

Another minor issue is the terminology describing Figure 2 in the text (p. 4, lines 94-102). Clta should be defined the first time it is used (line 96). In lines 98-99, both of the injected mRNAs are described as “(delta11)”, and as such should not be a positive control – I think the second should be “(11)”. The result that the fluorescence for the R26-mNG2(delta11)/+ embryos injected with Clta-mNG2(11) is less than the positive control should be included in the Results section and not just in the figure legend.

Thank you for these suggestions! We have revised the text accordingly (Lines 92-98). We have also removed the statement about relative fluorescence level from the legend since we do not want to overinterpret images captured by epifluorescence microscope.

Second decision letter

MS ID#: DEVELOP/2020/197418

MS TITLE: Efficient generation of endogenous protein reporters for mouse development

AUTHORS: Daniel O'Hagan and Amy Ralston

I have now received all the referees reports on the above manuscript, and have reached a decision. The referees' comments are appended below, or you can access them online: please go to BenchPress and click on the 'Manuscripts with Decisions' queue in the Author Area.

The overall evaluation is positive and we would like to publish your manuscript in Development. However as you'll see the reviewers have raised some very minor issues, which you might want to address before uploading the final manuscript (one is just a quick fix to a figure legend). If you do not agree with any of their criticisms or suggestions explain clearly why this is so. Your manuscript will not require any further review, rather I will accept it once the final version has been uploaded.

We are aware that you may currently be unable to access the lab to undertake experimental revisions. If it would be helpful, we encourage you to contact us to discuss your revision in greater detail. Please send us a point-by-point response indicating where you are able to address concerns raised (either experimentally or by changes to the text) and where you will not be able to do so within the normal timeframe of a revision. We will then provide further guidance. Please also note that we are happy to extend revision timeframes as necessary.

Reviewer 1

Advance summary and potential significance to field

n/a

Comments for the author

In the revised version of the manuscript, O’Hagan and Ralston addressed some of my concerns. However they still failed to sufficiently reply to my main criticism, namely the lack of side-by-side comparison of V5-based system signal alongside the antibody staining. I do appreciate that the Covid19 pandemic made finishing any experiments difficult and I do acknowledge that providing images that resemble a known pattern of expression has its value. Nevertheless, in the current form it is difficult to assess how fatefully the pattern of expression of various transcriptions factors, presented in the manuscript matches the endogenous protein distribution. For example, in Fig 5C the pattern of Nanog localisation is somehow surprising, since the signal is present in the ICM as well as in some TE cells. Although it is possible to see some Nanog in TE cells at this stage, usually the strength of the signal is higher in ICM cells. Providing the immunostaining alongside V5-

© 2021. Published by The Company of Biologists under the terms of the Creative Commons Attribution License (https://creativecommons.org/licenses/by/4.0/).

based signal array could clarify whether the two TE cells in this embryo indeed have higher Nanog levels (for whatever reason) or not. The same applies to V5-Gata3 signal. It seems like not all of the cells in the TE are Gata3-positive (which should have been the case at this stage). I do acknowledge the existence of variability between embryos at this stage and that perhaps not all TE cells in this particular embryo were Gata3-positive. However, without confirmation via immunostaining, it is impossible to judge.
Having said that, I believe that this manuscript describes an important technological development that could benefit other researchers in the field. Therefore, I leave it to the editor to decide whether due to the existing situation with Covid19 pandemic, the authors can be exempt from providing the additional information I asked for.

Reviewer 2

Advance summary and potential significance to field

The authors describe a useful method to endogenously tag proteins of interest with high knock-in efficiency. They have addressed all of my comments and I had one minor suggestion.

Comments for the author

The figure legend for Figure 3E is missing the information about the astrics (* and **) and I think there is a typo because this information is currently in the legend for Figure 3D, it just needs to be moved over.

Second revision

Author response to reviewers' comments

We include new data in the new Fig. S1, as requested by R1, and corrected the figure legend typo as requested by R2. Thank you!

Third decision letter

MS ID#: DEVELOP/2020/197418

MS TITLE: Efficient generation of endogenous protein reporters for mouse development

AUTHORS: Daniel O'Hagan, Robin E. Kruger, Bin Gu, and Amy Ralston
ARTICLE TYPE: Techniques and Resources Article

I am happy to tell you that your manuscript has been accepted for publication in Development, pending our standard ethics checks.

© 2021. Published by The Company of Biologists under the terms of the Creative Commons Attribution License (https://creativecommons.org/licenses/by/4.0/).
